# Morphology of the Myoepithelial Cell: Immunohistochemical Characterization from Resting to Motile Phase

**DOI:** 10.1100/2012/252034

**Published:** 2012-08-05

**Authors:** Germana Beha, Giuseppe Sarli, Barbara Brunetti, Francesco Sassi, Domenico Ferrara, Cinzia Benazzi

**Affiliations:** ^1^Department of Veterinary Medical Science, University of Bologna, 40064 Ozzano Emilia, Italy; ^2^Institute for Cancer Research and Treatment (IRCC), Fourth Floor, 10060 Candiolo, Italy

## Abstract

Myoepithelium is present in canine mammary tumors as resting and proliferative suprabasal and spindle and stellate interstitial cells. The aim of this paper was to evaluate a panel of markers for the identification of four different myoepithelial cell morphological types in the normal and neoplastic mammary gland and to investigate immunohistochemical changes from an epithelial to a mesenchymal phenotype. Cytokeratin 19 (CK19), cytokeratin 5/6 (CK5/6), cytokeratin 14 (CK14), estrogen receptor (ER), p63 protein, vimentin (VIM), and **α**-smooth muscle actin (Alpha-SMA) antibodies were used on 29 neoplasms (3 benign and 3 malignant myoepithelial tumors, 7 carcinomas in benign-mixed tumors and 16 complex carcinomas) and on normal tissue of mammary glands. All these antibodies were also tested on 3 mammary tissues from animals with no mammary pathology. The myoepithelial markers were well expressed in the suprabasal cells and gradually lost in the motile types, with the stellate cells maintaining only VIM expression typical of mesenchyma. ER labeled some resting and motile myoepithelial cells. On the basis of our results, we propose a transition from myoepithelial immotile cells into migratory fibroblast-like cells. This transition and the characterization of an immunohistochemical panel for resting and motile myoepithelial cells shed more light on the biological behavior of myoepithelial cells.

## 1. Introduction

Mammary gland tumors of dogs are formed by both epithelial (epithelium and myoepithelium) and mesenchymal components. The origin of the mesenchymal cells is still debated. The elevated frequency of tumors showing myoepithelial or basal cell proliferation is a unique feature of canine mammary tumors [1].

In the normal mammary gland, the lumina are delimitated by an inner layer of polarized epithelial cells resting on two outer or basal layers of epithelial and myoepithelial cells [2]. Both basal and myoepithelial cells synthesize the basement membrane of ducts and alveoli and form a structural barrier between the luminal epithelial cells and the surrounding stroma [3]. In ducts, myoepithelial cells form a nearly continuous layer of cells oriented parallel to the long axis of the ducts. This layer surrounds the luminal epithelial cells and separates them from the basement membrane and the stroma. In alveoli, the myoepithelial cells are discontinuous, forming a basket-like network around the alveoli, allowing some luminal epithelial cells to contact the basement membrane directly [3–5]. Therefore, the myoepithelium is not only located in an ideal position to communicate between these two compartments, but it is also positioned to provide important regulatory signals for the maintenance of normal cell structure [5].

Based on immunohistochemistry, the three layers of cells of the normal mammary gland display different markers: the luminal epithelium is labeled by CK19, and the basal cells and myoepithelial cells are stained by CK5/6 [6] and CK14 [2] and p63, Alpha-SMA, and VIM [2]. Myoepithelial cells are contractile elements exhibiting a combined epithelial and smooth muscle immunoprofile. The markers mentioned above are expressed in the cytoplasm, except for p63 which is a nuclear marker [1]. The myoepithelial cell layer is the sole source of tumor suppressor p63, which is significantly inhibited on proliferation and invasion of associated tumor cells [7]. In addition, basal myoepithelial cells in the normal mammary gland are occasionally labeled by ER antibody [8], which is used for the molecular-based classification of canine mammary tumors [9, 10].

 Distinct myoepithelial cell morphologies can be recognized in canine complex and mixed tumors: resting and proliferative suprabasal myoepithelial cells and spindle and stellate motile interstitial myoepithelial cells. Suprabasal cells are located between the basement membrane and the luminal epithelium and exhibit flattened spindle (resting cells) or polygonal morphologies (proliferative cells). Interstitial cells are frequently arranged in solid nests apposed to epithelial elements or isolated in the interstitium [1, 11]. Spindle and stellate myoepithelial cells differentiate toward a more general contractile phenotype [12].

Interstitial myoepithelial cells may eventually become fibroblast-like cells, showing only VIM immunoreactivity [11]. The myoepithelial differentiation may culminate in the formation of various mesenchymal tissues, including cartilage and bone in canine mammary mixed tumor.

The acquisition of typical features of mesenchymal cells is likely to originate through epithelial-mesenchymal transition (EMT). EMT is a biological phenomenon that allows a polarized epithelial cell, which normally interacts with the basement membrane via its basal surface, to undergo multiple biochemical changes enabling it to assume the traits and functions of mesenchymal cells [13].

This paper will focus on various aspects of myoepithelial cells and mammary tumors in dogs, specifically (1) characterization of the four different myoepithelial cell morphological types in the normal and neoplastic mammary gland using a panel of antibodies and (2) the immunohistochemical changes in myoepithelial cells from an epithelial to a mesenchymal phenotype.

## 2. Materials and Methods

### 2.1. Samples

Mammary gland specimens of 29 female dogs were retrieved from the database of the Anatomopathological Service of the Faculty of Veterinary Medicine of Bologna. The subjects belonged to different breeds: mongrel (*n* = 13), German shepherd (*n* = 3), Poodle (*n* = 3), Yorkshire Terrier (*n* = 3), Dachshund (*n* = 2), Setter (*n* = 1), Pointer (*n* = 1), Cocker spaniel (*n* = 1), Schnauzer (*n* = 1), and Siberian Husky (*n* = 1); they were all females, with an average age of 9.20 ± 2.28 years (mean ± SD). The tumors consisted of: 3 benign myoepithelial tumors, 3 malignant myoepithelial tumors, 7 carcinomas in benign mixed tumors, and 16 complex carcinomas (the last two groups were differentiated by the presence of cartilage and/or bone in the mixed tumors). In addition, 29 specimens from normal mammary glands of the same tumor line and 3 mammary samples from 3 healthy nonmammary tumor-bearing female dogs were evaluated.

Tumors were classified according to Misdorp et al. [14] and Goldschmidt et al. [15] into benign myoepithelial tumors: a rare neoplasm composed of myoepithelial cells arranged in short bundles admixed with an extracellular fibrillar basophilic material; malignant myoepithelial tumors: different from the benign variant with more polymorphic myoepithelial cells; complex carcinoma: a carcinoma composed of both luminal epithelial and myoepithelial components; carcinoma in benign tumor: a tumor with foci of malignant-appearing epithelial cells or distinct nodules of such cells occurring together with mesenchymal cells that have produced cartilage and/or bone possibly in combination with fibrous tissue.

### 2.2. Immunohistochemistry

Four *μ*m thick sections were cut from formalin-fixed paraffin-embedded blocks containing representative tumor samples. Immunohistochemistry for the following markers was done on these tissues: CK19, ER, CK5/6, CK14, VIM, Alpha-SMA, p63.

Sections were dewaxed in toluene and rehydrated. Endogenous peroxidase was blocked by immersion in 0.3% hydrogen peroxide for 20 min. Sections were then rinsed in Tris buffer. Antigen retrieval was performed with citrate buffer (2.1 g citric acid monohydrate/liter distilled water), pH 6.0 (except for CK5/6 and ER, which used EDTA, pH 8.0), and heating for two 5 min periods in a microwave oven at 750 W, followed by cooling at room temperature for 20 min. The primary antibodies are summarized in Table 1. All primary antibodies were incubated overnight at 4°C, followed by a commercial streptavidin-biotin-peroxidase technique (LSAB Kit, Dako, Amsterdam, The Netherlands). Diaminobenzidine (0.05% for 10 min at room temperature) was used as chromogen. Slides were counterstained with Papanicolaou's hematoxylin.

As a negative control, the primary antibody was replaced with an irrelevant, isotype-matched antibody to control for nonspecific binding of the secondary antibody. Positive tissue controls using the same IHC protocols included canine normal mammary gland (anti-CK19, -ER, -CK14, -VIM, Alpha-SMA, -p63 antibodies) and canine skin (anti-CK5/6).

The number of positive cells by each marker was calculated semiquantitatively: − = no stained cells, ± = less than 5% positive cells, + = 5–50% positive cells, ++ = more than 50% positive cells. Cases were considered positive for ER when nuclear staining was observed in at least 5% tumor cells [16].

## 3. Results

**Figure 1:**
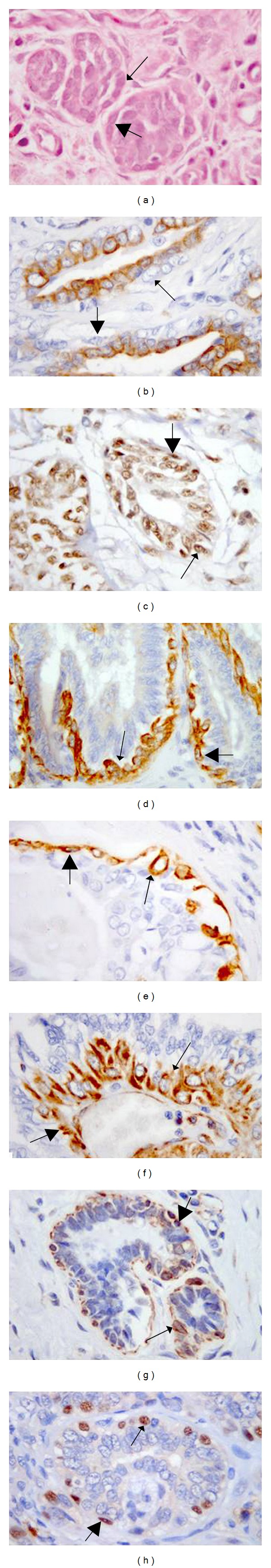
Suprabasal myoepithelial cells: resting (thin arrows) and proliferative (thick arrows) cells. Immunohistochemical expression of a panel of antibodies applied by IHC, 63x (a) Hematoxylin-eosin; (b) anti-CK19 antibodies labeling the cytoplasm; (c) anti-ER antibodies labeling the nuclei; (d) anti-CK 5/6 antibodies labeling the cytoplasm; (e) anti-CK14 antibodies labeling the cytoplasm; (f) anti-VIM antibodies labeling the cytoplasm; (g) anti-Alpha-SMA antibodies labeling the cytoplasmic membrane; (h) anti-p63 antibodies labeling the nuclei.

**Figure 2:**
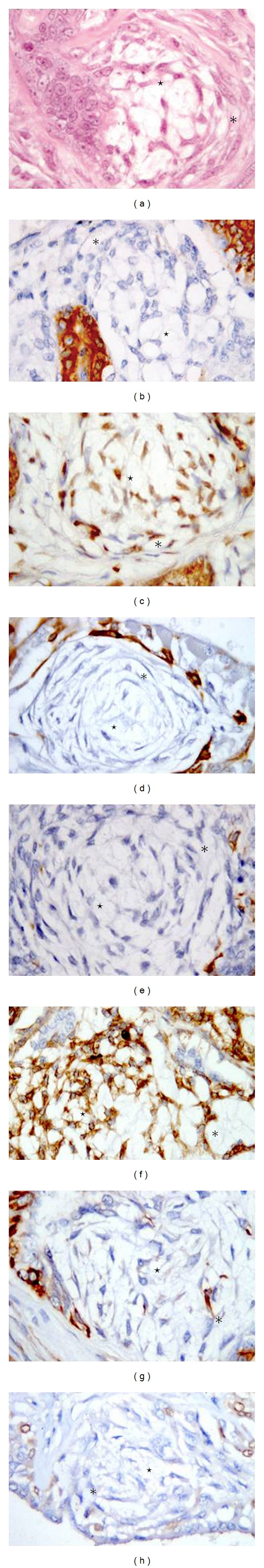
Motile myoepithelial cells: spindle (asterisks) and stellate (stars) cells. Immunohistochemical expression of a panel of antibodies applied by IHC. 63x (a) Hematoxylin-eosin; (b) anti-CK19 antibodies labeling the cytoplasm; (c) anti-ER antibodies labeling the nuclei; (d) anti-CK 5/6 antibodies labeling the cytoplasm; (e) anti-CK14 antibodies labeling the cytoplasm; (f) anti-VIM antibodies labeling the cytoplasm; (g) anti-Alpha-SMA antibodies labeling the cytoplasmic membrane; (h) anti-p63 antibodies labeling the nuclei.

Four types of myoepithelial cells were recognized on the basis of their morphology. The resting subtype exhibited the elongated features of spindle cells in close contact with luminal epithelial cells as well as proliferating suprabasal cells that instead showed a polygonal shape (Figure 1(a)). The interstitial motile cells were observed both forming nests (the spindle type lined nests and the stellate cells constituted the nest core) and isolated in the interstitium (Figure 2(a)).

### 3.1. Normal Mammary Gland

In the 3 control cases, all resting and proliferative suprabasal myoepithelial cells were labeled by p63, CK14, Alpha-SMA, and VIM. Resting and proliferative suprabasal myoepithelial cells did not express CK19 in any of the cases.

### 3.2. Mammary Tissue from the Same Line of the Mammary Tumor

In the 29 normal tissues in the same line as the tumors, all resting and proliferative suprabasal myoepithelial cells were labeled by p63, CK14, Alpha-SMA, and VIM. CK5/6 was positive in all but four cases and ER was detected in 12 cases. CK19 expression was only observed in the luminal epithelium. Myoepithelial motile interstitial cells were not observed. All the results are summarized in Table 2.

### 3.3. Mammary Tumors

Immunohistochemical results for suprabasal (Figure 1) and motile cells (Figure 2) using p63, CK14, CK5/6, CK19, Alpha-SMA, VIM, and ER are summarized in Table 2. CK5/6 labeled: suprabasal resting cells in 16 of the 29 cases (5 carcinomas in benign mixed tumors and 11 complex carcinomas). CK 5/6 also labeled proliferative suprabasal and spindle motile cells in 10 cases (3 carcinomas in benign mixed tumors and 7 complex carcinomas). Stellate motile cells were present in 25 cases (1 benign myoepithelial tumor, 3 malignant myoepithelial tumors, 5 carcinomas in benign mixed tumors and 15 complex carcinomas) but were negative for CK5/6. Cartilage in mammary mixed tumors was always negative.

CK14 showed positivity in 23 cases in resting cells (with the exception of 1 complex carcinoma and benign and malignant myoepithelial tumors), in 22 cases in proliferative cells (6 carcinomas in benign mixed tumors and 15 complex carcinomas) with a trend to lose the expression when these cells had acquired the more motile phenotype of spindle cells (positive in 2 benign and 3 malignant myoepithelial tumors, 2 carcinomas in benign mixed tumors and 2 complex carcinomas). Chondrocytes of mixed tumors were negative.

VIM was positive in suprabasal cells in the 23 cases of carcinoma in benign tumor and complex carcinoma and in motile myoepithelial cells in all 29 cases. Stromal cells were positive in all cases. Cartilage was VIM positive in all 7 carcinomas in benign-mixed tumors.

Alpha-SMA labeled resting and proliferative suprabasal myoepithelial cells in 23 carcinomas in benign-mixed tumors and complex carcinomas. The spindle cells in 10 cases (5 carcinomas in benign mixed tumor and 5 complex carcinomas) showed positivity for Alpha-SMA. In each case, except for two complex carcinomas, stellate cells were negative. Stroma showed positivity in only 6 cases (1 carcinoma in benign-mixed tumor and 5 complex carcinomas).

P63 was detected in resting and proliferative suprabasal myoepithelial cells of the 23 cases of carcinoma in benign-mixed tumor and complex carcinoma. All spindle and stellate motile cells were negative. Stromal cells and cartilage were negative.

ER expression was present in 11 suprabasal myoepithelial cells (4 carcinomas in benign-mixed tumors and 7 complex carcinomas); spindle and stellate motile cells were positive in 9 cases (3 carcinomas in benign-mixed tumors and 6 complex carcinomas). Cartilage of mixed tumors was negative.

Resting and proliferative suprabasal and spindle/stellate motile myoepithelial cells did not express CK19 in any of the tumors examined. Cartilage of mixed tumors was also negative for CK19.

## 4. Discussion

Based on the findings of Gama et al. [1] and Tateyama et al. [11], four morphological types of myoepithelial cells are present in the mammary gland: resting and proliferative suprabasal myoepithelial cells lining alveoli and ducts and spindle and stellate interstitial motile cells, which lie in the interstitial space where they may be arranged in nests. Myoepithelial markers, such as p63, CK5/6, CK14, Alpha-SMA, and VIM, proved to be valuable diagnostic adjuncts to facilitate the evaluation of complex and mixed proliferations. CK19 is considered the gold standard marker for luminal epithelium and was used to avoid any misdiagnosis with myoepithelial cells. Because of cross-reactivity patterns and the fact that lesional foci are typically minute, none of the myoepithelial markers enjoyed 100% sensitivity and specificity for myoepithelial cells. As such, at least 2 markers should be used to evaluate any given focus [17].

Based on our results, the best marker for suprabasal cells was p63 especially in association with CK14, which was limited to mature (basal) myoepithelial cells and, to a lesser extent followed by CK5/6, Alpha-SMA and VIM (Figure 1). However, CK5/6 also marked luminal epithelial cells making it difficult to distinguish them from proliferative suprabasal myoepithelial cells [2]. Morphologically, both epithelial and myoepithelial cells may have a polygonal shape. A characteristic of both CK14 and CK5/6, but not of p63, Alpha-SMA, and VIM, was their reduced expression in myoepithelial cells in the suprabasal proliferative state. CK14, CK5/6, and p63 expression was gradually lost in cells in the spindle and stellate motile state.

Alpha-SMA and VIM were present in spindle motile myoepithelial cells with different degrees of intensity. Only VIM proved to be a consistent marker for stellate motile myoepithelial cells. In this study, the stellate motile myoepithelium was arranged in nests and lined by resting cells presumably of alveolar origin. This feature may support the idea that the nests of stellate motile myoepithelial cells, which have lost expression of the main myoepithelial suprabasal markers, but retained affinity for VIM, are the precursors of cartilage, indicating that these cells have completed their transformation into mesenchymal elements. In benign and malignant myoepithelial tumors, VIM labeling in all cases, loss of all other suprabasal myoepithelial markers, and the scant positivity to CK14 in spindle cells were indicative of a prevailing expression of the myoepithelium motile state and a possible passage from simple myoepithelial cells to mesenchymal fibroblasts.

In our study, evidence of the myoepithelial cells shifting to a mesenchymal phenotype, shown by the loss of CK14, CK5/6, and p63 expression, was reinforced by the discontinuous labeling of spindle cells for Alpha-SMA, a marker of both myoepithelial cells and myofibroblasts, which was completely lost in stellate motile cells that have supposedly become fibroblasts.

Further confirmation studies by Tsuda et al. [18] reported the occurrence of myofibroblasts with remnants of CK14 expression (described as “converted myoepithelial cells”). In the cases examined in the present study, CK14 progressively faded, therefore indicating a loss of the (myo-)epithelial phenotype. These results support the EMT hypothesis involving a myoepithelial-like state [19], which undergoes a myoepithelial mesenchymal transition (MMT). This hypothesis was confirmed in the dog by Gärtner et al. [20] who stated that in mammary tumors one of the steps in the evolution of mesenchymal cells involves the expression of typical myoepithelial traits.

An interesting result of the present study was the positivity to ER found in 12/29 suprabasal myoepithelial cells and 9/29 stellate cells of carcinoma in benign-mixed tumors and complex carcinomas. Two isoforms of ER receptors have been described, namely, ER-*α* and ER-*β*, the latter being the only form expressed in the nuclei of isolated basal-myoepithelial cells [8]. The antibody used in the present investigation was inclusive of both isoforms: both luminal and basal/stellate cells were labeled, presumably luminal cells by ER-*α* and basal/stellate cells by ER-*β*.

In conclusion, the suprabasal myoepithelial cells were well characterized by p63 and CK14 and to a lesser extent by the other marker used. The motile myoepithelial cells are instead characterized by Alpha-SMA and VIM and loss of CK14, CK5/6, and p63 (Figure 2). The present study also demonstrated ER in both luminal epithelial and suprabasal/stellate myoepithelial cells (the latter in about half of the cases) and that ER expression is not influenced by the resting/motile phase. Therefore, in serial or multistained sections, immunohistochemistry to ER in combination with p63 and CK14 may serve to avoid erroneous identification of luminal or myoepithelial cells in canine mammary tumors. The trend of preserved Alpha-SMA and VIM expression in spindle cells, and only VIM positivity in stellate motile cells as well as the decreased p63 expression in both motile types, supports the hypothesis of the EMT involving a myoepithelial-like state [19] in MMT. The spindle motile cell could be considered an earlier transformation than the stellate cell towards a mesenchymal phenotype.

## Supplementary Material

Supplementary Table 1: Immunohistochemical results.
Supplementary Table 2: Age, Breed and Clinical Findings.

## Figures and Tables

**Table 1:** Primary antibodies, resources, and dilutions used in immunohistochemistry.

Antibody (anti-)	Clone	Manufacturer	Dilution
P63	4A4	Dako (Glostrup, Denmark)	1 : 50
Alpha-SMA	1A4	Dako (Glostrup, Denmark)	1 : 100
Cytokeratin 19	BA17	Dako (Glostrup, Denmark)	1 : 50
Cytokeratin 14	Ab-1 (LL002)	NeoMarkers (Fremont, CA, USA)	1 : 300
Cytokeratins 5/6	D5/16B4	Zymed (South San Francisco, CA, USA)	1 : 100
VIM	V9	Dako (Glostrup, Denmark)	1 : 100
ER	1D5	Dako (Glostrup, Denmark)	1 : 25

**Table 2:** Immunohistochemical results for suprabasal and motile myoepithelial cells.

Type of lesion	Cell morphology					Antibodies for^∗^			
			p63	CK14	CK5/6	CK19	Alpha-SMA	VIM	ER
Normal mammary gland (*n* = 3)^°^	Suprabasal	resting	++	++	+	−	++	++	+
		proliferative	++	+	+	−	++	++	+

Mammary tissue in the same tumor line (*n* = 29)^°^	Suprabasal	resting	++	++	++	−	++	++	+(12/29)
		proliferative	++	+	+	−	++	++	+(12/29)

Benign myoepithelioma (*n* = 3)^§^	Motile	spindle	−	±(2/3)	−	−	−	++	−
		stellate	−	−	−	−	−	++	−

Malignant myoepithelioma (*n* = 3)^§^	Motile	spindle	−	±	−	−	−	++	−
		stellate	−	−	−	−	−	++	−

Carcinoma in benign-mixed tumor (*n* = 7)	Suprabasal	resting	++	++	+(5/7)	−	++	++	+(4/7)
		proliferative	++	+/±(2/7)	+(3/7)	−	++	++	+(4/7)
	Motile	spindle	−	±(2/7)	+(3/7)	−	±(5/7)	++	+(3/7)
		stellate	−	−	−	−	−	++	+(3/7)

Complex carcinoma (*n* = 16)	Suprabasal	resting	++	++(15/16)	+(11/16)	−	++	++	+(7/16)
		proliferative	++	+(15/16)	+(7/16)	−	++	++	+(7/16)
	Motile	spindle	−	±(2/16)	+(7/16)	−	±(5/16)	++	+(6/16)
		stellate	−	−	−	−	±(2/16)	++	+(6/16)

^∗^−: no stained cells; ±: less than 5% positive cells; +: 5–50% positive cells; ++: more than 50% positive cells.

^°^: the motile phonotype is not updated because not present.

^§^: the suprabasal phonotype is not updated because not detectable around luminal cells.
