# Supplementary material for: Morphology of the Myoepithelial Cell: Immunohistochemical Characterization from Resting to Motile Phase
Source: ScientificWorldJournal. 2012 Aug 5;2012:252034. doi: 10.1100/2012/252034 (PMC3420080; doi:10.1100/2012/252034)
Supplement: Supplementary file 1 — Supplementary Table 1: Immunohistochemical results. Supplementary Table 2: Age, Breed and Clinical Findings. [file 252034.f1.docx]

**Supporting Table 1: Immunohistochemical results**

| **Type of lesion** | **Case ID** | **Cell morphology** | **Antibodies*** | | | | | | |
| --- | --- | --- | --- | --- | --- | --- | --- | --- | --- |
|  |  |  | **p63** | **CK14** | **CK5/6** | **CK19** | **Alpha-SMA** | **VIM** | **ER** |
| **Benign myoepithelioma°** | 1722/01 | spindle | - | - | - | - | - | ++ | - |
|  |  | stellate | - | - | - | - | - | ++ | - |
|  | 712/01 | spindle | - | ± | - | - | - | ++ | - |
|  |  | stellate | - | - | - | - | - | ++ | - |
|  | 1506/01 | spindle | - | ± | - | - | - | ++ | - |
|  |  | stellate | - | - | - | - | - | ++ | - |
| **Malignant myoepithelioma°** | 358/01 | spindle | - | ± | - | - | - | ++ | - |
|  |  | stellate | - | - | - | - | - | ++ | - |
|  | 571/01 | spindle | - | ± | - | - | - | ++ | - |
|  |  | stellate | - | - | - | - | - | ++ | - |
|  | 659/01 | spindle | - | ± | - | - | - | ++ | - |
|  |  | stellate | - | - | - | - | - | ++ | - |
| **Carcinoma in benign mixed tumour** | 32140 | resting | ­­++ | ++ | + | - | ++ | ++ | - |
|  |  | proliferative | ++ | - | + | - | ++ | ++ | - |
|  |  | spindle | - | - | + | - | - | ++ | - |
|  |  | stellate | - | - | - | - | - | ++ | - |
|  | 34382 | resting | ++ | ++ | + | - | ++ | ++ | - |
|  |  | proliferative | ++ | - | - | - | ++ | ++ | - |
|  |  | spindle | - | - | - | - | ± | ++ | - |
|  |  | stellate | - | - | - | - | - | ++ | - |
|  | 34105 | resting | ++ | ++ | - | - | ++ | ++ | + |
|  |  | proliferative | ++ | - | - | - | ++ | ++ | + |
|  |  | spindle | - | - | - | - | ± | ++ | + |
|  |  | stellate | - | - | - | - | - | ++ | + |
|  | 33765 | resting | ++ | ++ | + | - | ++ | ++ | + |
|  |  | proliferative | ++ | + | + | - | ++ | ++ | + |
|  |  | spindle | - | ± | + | - | ± | ++ | - |
|  |  | stellate | - | - | - | - | - | ++ | - |
|  | 31842 | resting | ++ | ++ | + | - | ++ | ++ | - |
|  |  | proliferative | ++ | - | - | - | ++ | ++ | - |
|  |  | spindle | - | - | - | - | ± | ++ | - |
|  |  | stellate | - | - | - | - | - | ++ | - |
|  | 34405 | resting | ++ | ++ | + | - | ++ | ++ | + |
|  |  | proliferative | ++ | - | + | - | ++ | ++ | + |
|  |  | spindle | - | - | + | - | ± | ++ | + |
|  |  | stellate | - | - | - | - | - | ++ | + |
|  | 34104 | resting | ++ | ++ | - | - | ++ | ++ | + |
|  |  | proliferative | ++ | ± | - | - | ++ | ++ | + |
|  |  | spindle | - | ± | - | - | - | ++ | + |
|  |  | stellate | - | - | - | - | - | ++ | + |
| **Complex carcinoma** | 29504 | resting | ++ | - | + | - | ++ | ++ | + |
|  |  | proliferative | ++ | - | + | - | ++ | ++ | + |
|  |  | spindle | - | - | + | - | - | ++ | + |
|  |  | stellate | - | - | - | - | - | ++ | + |
|  | 31194 | resting | ++ | ++ | + | - | ++ | ++ | + |
|  |  | proliferative | ++ | + | + | - | ++ | ++ | + |
|  |  | spindle | - | - | + | - | - | ++ | + |
|  |  | stellate | - | - | - | - | - | ++ | + |
|  | 33402 | resting | ++ | ++ | + | - | ++ | ++ | - |
|  |  | proliferative | ++ | + | - | - | ++ | ++ | - |
|  |  | spindle | - | ± | - | - | - | ++ | - |
|  |  | stellate | - | - | - | - | - | ++ | - |
|  | 33664 | resting | ++ | ++ | - | - | ++ | ++ | + |
|  |  | proliferative | ++ | + | - | - | ++ | ++ | + |
|  |  | spindle | - | - | - | - | - | ++ | + |
|  |  | stellate | - | - | - | - | - | ++ | + |
|  | 33723 | resting | ++ | ++ | + | - | ++ | ++ | - |
|  |  | proliferative | ++ | + | + | - | ++ | ++ | - |
|  |  | spindle | - | - | + | - | - | ++ | - |
|  |  | stellate | - | - | - | - | - | ++ | - |
|  | 34251 | resting | ++ | ++ | + | - | ++ | ++ | + |
|  |  | proliferative | ++ | + | + | - | ++ | ++ | + |
|  |  | spindle | - | - | + | - | ± | ++ | + |
|  |  | stellate | - | - | - | - | - | ++ | + |
|  | 36260 | resting | ++ | ++ | - | - | ++ | ++ | - |
|  |  | proliferative | ++ | + | - | - | ++ | ++ | - |
|  |  | spindle | - | - | - | - | ± | ++ | - |
|  |  | stellate | - | - | - | - | ± | ++ | - |
|  | 34567 | resting | ++ | ++ | + | - | ++ | ++ | + |
|  |  | proliferative | ++ | + | + | - | ++ | ++ | + |
|  |  | spindle | - | - | + | - | ± | ++ | - |
|  |  | stellate | - | - | - | - | - | ++ | - |
|  | 33766 | resting | ++ | ++ | + | - | ++ | ++ | - |
|  |  | proliferative | ++ | + | - | - | ++ | ++ | - |
|  |  | spindle | - | - | - | - | - | ++ | - |
|  |  | stellate | - | - | - | - | - | ++ | - |
|  | 31509 | resting | ++ | ++ | + | - | ++ | ++ | - |
|  |  | proliferative | ++ | + | - | - | ++ | ++ | - |
|  |  | spindle | - | - | - | - | ± | ++ | - |
|  |  | stellate | - | - | - | - | - | ++ | - |
|  | 34516 | resting | ++ | ++ | + | - | ++ | ++ | + |
|  |  | proliferative | ++ | + | - | - | ++ | ++ | + |
|  |  | spindle | - | - | - | - | - | ++ | + |
|  |  | stellate | - | - | - | - | - | ++ | + |
|  | 30746 | resting | ++ | ++ | - | - | ++ | ++ | + |
|  |  | proliferative | ++ | + | - | - | ++ | ++ | + |
|  |  | spindle | - | - | - | - | - | ++ | + |
|  |  | stellate | - | - | - | - | - | ++ | + |
|  | 31777 | resting | ++ | ++ | - | - | ++ | ++ | - |
|  |  | proliferative | ++ | + | - | - | ++ | ++ | - |
|  |  | spindle | - | - | - | - | - | ++ | - |
|  |  | stellate | - | - | - | - | - | ++ | - |
|  | 32992 | resting | ++ | ++ | + | - | ++ | ++ | - |
|  |  | proliferative | ++ | + | + | - | ++ | ++ | - |
|  |  | spindle | - | - | + | - | - | ++ | - |
|  |  | stellate | - | - | - | - | - | ++ | - |
|  | 29855 | resting | ++ | ++ | - | - | ++ | ++ | - |
|  |  | proliferative | ++ | + | - | - | ++ | ++ | - |
|  |  | spindle | - | ± | - | - | - | ++ | - |
|  |  | stellate | - | - | - | - | - | ++ | - |
|  | 28640 | resting | ++ | ++ | + | - | ++ | ++ | - |
|  |  | proliferative | ++ | + | + | - | ++ | ++ | - |
|  |  | spindle | - | - | + | - | ± | ++ | - |
|  |  | stellate | - | - | - | - | ± | ++ | - |

* - = no stained cells; ± = less than 5% positive cells; + = 5 - 50% positive cells;

++ = more than 50% positive cells

° = the motile phonotype is not updated because not present

§ = the suprabasal phonotype is not updated because no detectable around luminal cells

**Supporting Table 2: Age, Breed and Clinical Findings**

| **CASE ID** | | **TYPE OF LESION** | **BREED** | **AGE (YEARS)** | **CLINICAL FINDINGS** | |
| --- | --- | --- | --- | --- | --- | --- |
|  |  |  |  |  | **ANATOMICAL SITE°** | **MEASUREMENTS (cm)** |
| **1** | 1722/01 | Benign myoepithelioma | Poodle | 6 | 5L | 4 |
| **2** | 712/01 | Benign myoepithelioma | Mixed breed | 8 | 5L | 3 |
| **3** | 1506/01 | Benign myoepithelioma | Poodle | 12 | 1R | NA |
| **4** | 358/01 | Malignant myoepithelioma | Siberian husky | 14 | 3R | 2 |
| **5** | 571/01 | Malignant myoepithelioma | Mixed breed | 11 | 2L | NA |
| **6** | 659/01 | Malignant myoepithelioma | German shepherd | 12 | 5L | NA |
| **7** | 32140 | Carcinoma in benign mixed .tumor | Mixed breed | 9 | 2S | 0.5 x 1 |
| **8** | 34382 | Carcinoma in benign mixed tumor | Mixed breed | 8 | 5R | 5 x 6 |
| **9** | 34105 | Carcinoma in benign mixed tumor | Cocker | 4 | 2R | 1 |
| **10** | 33765 | Carcinoma in benign mixed tumor | Dachshund | 10 | 2L | 4 |
| **11** | 31842 | Carcinoma in benign mixed tumor | German shepherd | 8 | 4R | 2.5 |
| **12** | 34405 | Carcinoma in benign mixed tumor | Mixed breed | 11 | 5L | 2 |
| **13** | 34104 | Carcinoma in benign mixed tumor | Yorkshire | 7 | 2L | NA |
| **14** | 29504 | Complex carcinoma | Setter | 7 | 5R | 3 x 2 |
| **15** | 31194 | Complex carcinoma | Mixed breed | 9 | 5R | 10 x 4 |
| **16** | 33402 | Complex carcinoma | Dachshund | 7 | 3R | 1 |
| **17** | 33664 | Complex carcinoma | German shepherd | 10 | 3R | 4 |
| **18** | 33723 | Complex carcinoma | Mixed breed | 13 | 4R | 1 |
| **19** | 34251 | Complex carcinoma | Mixed breed | 9 | 5R | 7 x 5 |
| **20** | 36260 | Complex carcinoma | Pointer | 10 | 4L | 14 x 8 |
| **21** | 34567 | Complex carcinoma | Schnautzer | 6 | 5R | NA |
| **22** | 33766 | Complex carcinoma | Mixed breed | 12 | 5R | 7 x 5 |
| **23** | 31509 | Complex carcinoma | Mixed breed | 10 | 4R | NA |
| **24** | 34516 | Complex carcinoma | Mixed breed | 8 | 3R | 0.5 |
| **25** | 30746 | Complex carcinoma | Poodle | 7 | 5L | NA |
| **26** | 31777 | Complex carcinoma | Yorkshire | 11 | 1R | 0.8 |
| **27** | 32992 | Complex carcinoma | Yorkshire | 9 | 5L | 3 x 4 |
| **28** | 29855 | Complex carcinoma | Mixed breed | 10 | 5L | 3 x 2 |
| **29** | 28640 | Complex carcinoma | Mixed breed | 9 | 5R | 4 x 5 |

**°**Anatomical site: 1L: left thoracic gland, 1R: right thoracic gland, 2L: left cranial abdominal gland, 2R: right cranial abdominal gland, 3L: left caudal abdominal gland, 3R: right caudal abdominal gland, 4L: left cranial inguinal gland, 4R: right cranial inguinal gland, 5L: left caudal inguinal gland, 5R: right caudal inguinal gland. NA: not available
